# Attaining Base Camp: Moving in the Right Direction, Miles Still to Go

**DOI:** 10.1212/NE9.0000000000200036

**Published:** 2022-12-09

**Authors:** Catherine S.W. Albin

**Affiliations:** From the Department of Neurology, Emory University School of Medicine, Atlanta, GA.

Simulation is a highly engaging methodology for both teaching and assessment. Yet simulation frameworks, techniques, and procedures have largely been developed and investigated outside of the neurologic context, and much work remains in understanding how simulation can be best leveraged by neurology educators. In this issue of *Neurology*® *Education*, Tchopev et al. and Pergakis et al. report important findings from 2 acute neurology simulation curricula.^1,2^

In both studies, the learners found the experience meaningful and engaging, but beyond the shared enthusiasm of participants, these studies represent 2 disparate points on the continuum of how simulation can be applied in neurology. In the study by Tchopev et al., simulation is explored as an engaging method for teaching in a safe learning environment—simulation as a formative experience. In the study by Pergakis et al., simulation is used to assess trainee performance—simulation as a summative experience. Both provide continued evidence of the feasibility and educational value of simulation, with the study by Pergakis et al. also highlighting how we can establish validity. Both, however, underscore the heights we have yet to ascend to create evidence-based best practices for our field (Figure).

**Figure:**
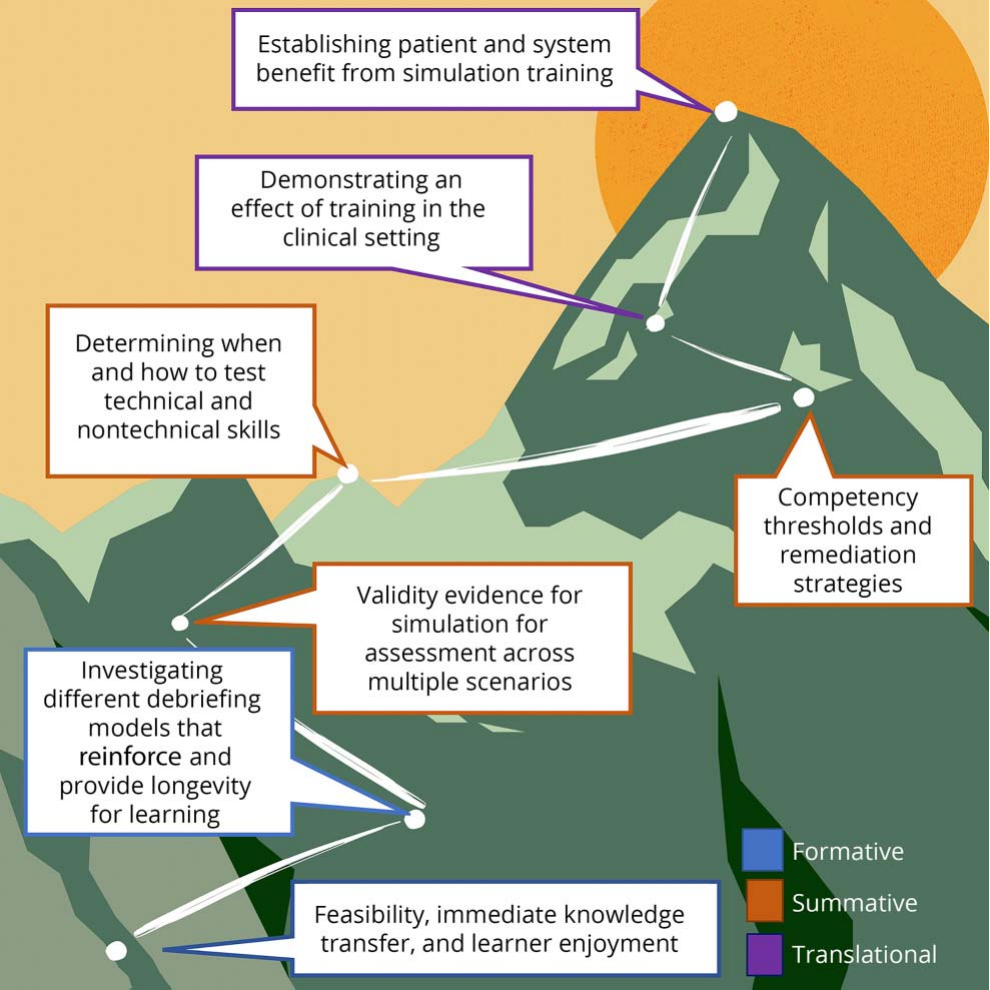
Simulation From Base Camp to Summit A representation of the steps necessary to demonstrate how simulation can be used for assessment and ultimately to have an effect on patient and health system outcomes.

## Simulation as a Formative Experience

Tchopev et al. used self-assessments and a limited knowledge assessment before and after the curriculum to demonstrate learning. As expected, students performed better on most knowledge questions and reported higher confidence after the simulation. This work establishes that learners react well to simulation, but it invites many more questions—how can we assess long-term retention and bedside effect of simulation curriculum? As the authors note, self-assessment is subject to bias and is a low-level outcome in Kirkpatrick's model of curricular assessment.^3,4^ Improvement in knowledge-based questions provides an indication of factual acumen but may not translate to management at the bedside. Equally important to *how* we should measure learning is *when* we should measure it. Assessments delivered directly after a simulation measure only short-term learning. Evaluating outcomes too far removed from the simulation may confound the assessment with additional learning, maturation, or skill decay.

Furthermore, many of these formative curricula are used to develop “nontechnical” skills such as communication, triage, teamwork, and cognitive load reduction. These attributes are difficult to measure but are equally important in the practice of medicine. It will be important to determine when nontechnical skills should be emphasized or if there needs to be separate technical and nontechnical training. Directly assessing these skills, providing feedback to trainees, and conducting follow-up observation in a clinical setting may be needed to understand how practice of nontechnical skills improves bedside performance.

Finally, while the actual simulation is important in activating limbic pathways and engaging learners in decision-making, the formative experience of simulation is debriefing.^5-8^ Any study of simulation as a formative experience needs to explore the style and lens through which debriefing is accomplished. Educators can benefit from training in these techniques (such as Debriefing with Good Judgement,^9^ TeamGAINS,^10^ PEARLS,^11^ GIFT,^12^ etc.). Simulation studies should clearly define the approach to debriefing and the framework used (Figure).

## Simulation as a Summative Experience

With the Accreditation Council for Graduate Medical Education milestones revision,^13^ there has been an emphasis on moving toward more objective methods to measure trainee performance. Pioneering simulation work has demonstrated that for status epilepticus, achieving a “ready to graduate” milestone had poor predictive value for how well they performed in a status epilepticus simulation.^14^ The study by Pergakis et al. provides further important validity evidence for simulation in acute neurology. By having many different levels of learners complete the same simulation case, they demonstrate that the trainees' level of experience had a significant effect on the critical action sum score (improving with increasing experience), suggesting that simulation can be used to benchmark achievement. Future studies have much to build on. If simulation is going to be used for evaluation, who sets the competency bar? Scores must include attaining technical milestones, but should we also aim to assess nontechnical skills: how well does the trainee triage, integrate new information, and provide closed-loop communication? If so, what are those benchmarks?

We will also need to qualitatively assess why trainees make mistakes. Pergakis et al. provide an important window into the mental frameworks of trainees: many had anchoring bias that prevented them from reimaging the situation as an infection. In this way, simulation uncovers 2 different kinds of mistakes: (1) “I didn't remember correctly” (best categorized as a low-level mistake in Blooms Taxonomy, e.g., the trainee forgot to give thiamine) and (2) “I didn't analyze correctly” (a higher-level Blooms Taxonomy mistake, e.g., the trainee failed to reconsider an alternative etiology of status). While the lower-level mistakes can be remedied by checklists and cognitive aids, the higher-level analytic mistake requires coaching trainees to avoid cognitive missteps; to essentially “think better.” This—understanding how trainee's think—is perhaps the most interesting application of simulation and as such deserves dedicated study. Certainly, remedying narrowed thinking or failed heuristics may not be possible over 1 simulation, but a thoughtful debrief may provide trainees with insight about their own bias and blind spots. We should assess the degree to which it does.

Finally, and of course most challengingly, educators must demonstrate that simulation results in patient-level outcome benefits. Demonstrating that central line training reduced central line–associated blood stream infections and saved healthcare dollars set the gold standard for simulation as a translational science.^15^ While we have evidence that simulation may improve door to needle times in acute stroke,^16^ we have yet to explore how simulation of procedural training might translate into tangible quality improvements in the bedside (e.g., brain death testing or endovascular stroke training). To demonstrate these higher Kirkpatrick feats will require multicenter collaboration and rigorous study.

For simulation in neurology, it is clear the steepest ascent is ahead. Undoubtably, though, there are educators up for the challenge.
